# Extreme active matter at high densities

**DOI:** 10.1038/s41467-020-16130-x

**Published:** 2020-05-22

**Authors:** Rituparno Mandal, Pranab Jyoti Bhuyan, Pinaki Chaudhuri, Chandan Dasgupta, Madan Rao

**Affiliations:** 1 Simons Centre for the Study of Living Machines, National Centre for Biological Sciences (TIFR), Bangalore, 560065 Karnataka India; 2 Centre for Condensed Matter Theory, Department of Physics, Indian Institute of Science, Bangalore, 560012 Karnataka India; 3 The Institute of Mathematical Sciences, Chennai, 600113 Tamil Nadu India; 4 International Centre for Theoretical Sciences (TIFR), Bangalore, 560089 Karnataka India

**Keywords:** Biological physics, Statistical physics

## Abstract

We study the remarkable behaviour of dense active matter comprising self-propelled particles at large Péclet numbers, over a range of persistence times, from *τ*_*p*_ → 0, when the active fluid undergoes a slowing down of density relaxations leading to a glass transition as the active propulsion force *f* reduces, to *τ*_*p*_ → *∞*, when as *f* reduces, the fluid jams at a critical point, with stresses along force-chains. For intermediate *τ*_*p*_, a decrease in *f* drives the fluid through an intermittent phase before dynamical arrest at low *f*. This intermittency is a consequence of periods of jamming followed by bursts of plastic yielding associated with Eshelby deformations. On the other hand, an increase in *f* leads to an increase in the burst frequency; the correlated plastic events result in large scale vorticity and turbulence. Dense extreme active matter brings together the physics of glass, jamming, plasticity and turbulence, in a new state of driven classical matter.

## Abstract (web summary)

While active matter exhibits unusual dynamics at low density, high density behavior has not been explored. Mandal et al. show that extreme dense active matter, shows a rich spectrum of behaviour from intermittent plastic bursts and turbulence, to glassy states and jamming in the limit of infinite persistence time.

## Introduction

Active matter, where each particle comprising the system is driven by an internal energy source and dissipates it in movement, constitutes a new class of driven nonequilibrium systems^1^. Extreme active matter, where the magnitude of the propulsion force *f* is higher than inter-particle or thermal forces, and the direction of the propulsion force persists over times *τ*_*p*_ longer than characteristic relaxation times of the system in the absence of activity, is an extreme realisation of activity. In this limit, active systems must show strong departures from equilibrium; this expectation is borne out in active Ornstein–Uhlenbeck particles (AOUPs) at low densities^2^, where steady states with finite energy–current manifest when the persistence time is sufficiently large. Even so, one might suspect that at very high densities, these distinguishing effects of activity will be firmly suppressed^3–14^. On the contrary, we will see that extreme active matter at high densities is a fount of surprises, bringing together the physics of glass, jamming, plasticity and turbulence, in a new state of driven classical matter. Towards the end, we will discuss possible realisations, both natural and synthetic, of such dense extreme active matter.

**Fig. 1:**
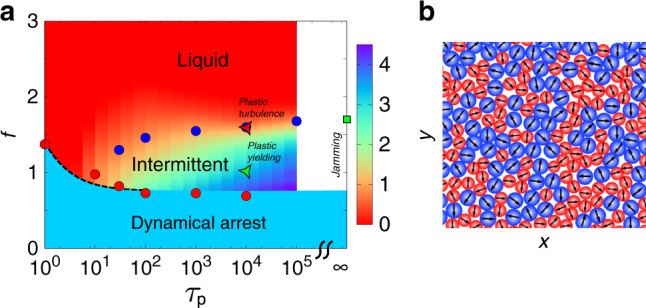
**Dynamical phase diagram in the***f* − *τ*_*p*_**plane at fixed density**. **a** At low *τ*_*p*_ (0 < *τ*_*p*_ < 10), there is a direct transition at *f* = *f*_*c*_(*τ*_*p*_) (red dots), from the liquid to a dynamically arrested phase, as measured from the the divergence of *τ*_*α*_, the slow relaxation of the density fluctuations (Fig. 2c). This transition to the dynamically arrested state continues into larger values of *τ*_*p*_ (red dots). The dashed line is a fit to the active RFOT theory^16^, with an effective temperature *Af*^ 2^*τ*_*p*_/(1 + *Bτ*_*p*_), where *A* and *B* are fit parameters. At larger values of *τ*_*p*_ > 10, a new intermittent phase intervenes between the liquid and the dynamically arrested phases. The liquid-intermittent phase boundary, *f* = *f*^ *^(*τ*_*p*_), is obtained from the dynamical order parameter, the excess kurtosis *κ*_*ex*_ (Fig. 2e) and the peak height *h*_*p*_ (Fig. 2f), shown with blue circles. This transition is very sharp and continuous at large values of *τ*_*p*_, and gets progressively broader and less defined at lower values of *τ*_*p*_ (colour bar represents the value of *κ*_*ex*_(0^+^) in ${\mathrm{log}\,}_{10}$ for different *f* and *τ*_*p*_). This intermittent phase is characterised by bursts of plastic yielding and turbulence close to the transition to the liquid phase. At *τ*_*p*_ = *∞*, the assembly shows a sudden transition from a liquid to a jammed configuration at a force threshold, *f* = *f*^ *^(*∞*), where the particles suddenly get into a force-balanced configuration (Fig. 5d). **b** Schematic of a dense assembly of the bidisperse *A*(red)–*B*(blue) soft Lennard–Jones particles, with arrows marking the instantaneous direction of self-propulsion.

Our main results are summarised in Fig. 1: (i) for small values of *τ*_*p*_, the assembly smoothly transforms from a fluid at high *f* to a dynamically arrested glass at low *f*. The phase boundary is well described by an active generalisation of RFOT theory^15–19^, with an “effective temperature” that goes as *Af*^ 2^*τ*_*p*_/(1 + *Bτ*_*p*_), where *A* and *B* are constants. However, we find that the mean kinetic energy has a different scaling behaviour with *τ*_*p*_, pointing to the feature that active systems can not be characterised by a single effective temperature. (ii) At intermediate values of *τ*_*p*_, the fluid abruptly transforms into an intermittent fluid, characterised by intermittency in the kinetic energy, as *f* is lowered to *f*^ *^(*τ*_*p*_). The intermittency increases as *f* is reduced, until at low enough *f*, the assembly undergoes complete dynamical arrest. (iii) This intermittency is a consequence of periods of jamming followed by bursts of plastic yielding. We identify isolated plastic events with Eshelby deformations^20^, akin to the response of dense amorphous solids to an externally imposed shear. As one approaches *f*^ *^(*τ*_*p*_) from below, the plastic events become numerous and correlated in space–time, with an avalanche-like scale-invariant statistics, that results in vorticity and turbulence, characterised by an inverse cascade with a Kolmogorov exponent^21^. (iv) The accumulated yielding over a time window involves the cooperative movement of a finite fraction of particles, and should result in a viscoelastic fluid at large timescales. (v) In the limit *τ*_*p*_ → *∞*, the fluid reaches a jammed state on lowering *f* to *f*^ *^(*∞*), with stresses concentrated along force chains. As one moves away from this jamming threshold by decreasing *τ*_*p*_, the force chains dynamically remodel. The lifetime of force-balanced configurations diverges as one approaches the jamming point by increasing *τ*_*p*_ at fixed *f* = *f*^ *^(*∞*), with an exponent *z* ≈ 0.71.

## Results

### Model equations

These results, which we expect are generic to dense extreme active matter, have been obtained from a numerical study of the stochastic dynamics of a two-dimensional Kob–Andersen binary mixture^22^ of soft-interacting active brownian particles^23^, each of mass *m*, and driven by a stochastic self-propulsion force **f** = *f***n** whose direction ${\bf{n}}\equiv \left(\cos \theta ,\sin \theta \right)$ undergoes rotational diffusion. The equations of motion of the particles are given by

$$m{\ddot{{\bf{x}}}}_{i}=-\gamma {\dot{{\bf{x}}}}_{i}+\mathop{\sum }\limits_{i\ne j = 1}^{N}{{\bf{f}}}_{ij}+f{{\bf{n}}}_{i}+{\boldsymbol{\vartheta} }_{i},$$

1 $${\dot{\theta }}_{i}={\xi }_{i}.$$

where the *i*th particle is subject to a friction *γ* and a thermal noise ***ϑ***_*i*_ with zero mean and variance $2{k}_{B}T\gamma \delta (t-t^{\prime} )$ that obeys fluctuation–dissipation relation. The rotational diffusion of the orientation of the propulsion force *θ*_*i*_ is described by an athermal noise *ξ*_*i*_, with zero mean and correlation $\langle {\xi }_{i}(t){\xi }_{j}(t^{\prime} )\rangle =2{\tau }_{p}^{-1}{\delta }_{ij}\delta (t-t^{\prime} )$. Its effect on the **x**_*i*_ dynamics is as an exponentially correlated vectorial noise with correlation time *τ*_*p*_, which being unrelated to the drag *γ*, violates fluctuation–dissipation relation. The inter-particle force **f**_*ij*_ is modelled via Lennard–Jones (LJ) interaction with particle diameter *σ* (see “Methods”). Here, we have fixed the number density to be 1.2, which is in the regime where this Kob–Andersen model^22^ of binary mixture of passive soft spheres shows dynamical arrest at low temperatures. In our study, we focus on the strictly athermal limit *T* = 0; we have checked that our results hold when *T* is small. Further, we choose *γ* = 1, leading to the particle inertial relaxation time ${\tau }_{\gamma }\equiv \frac{m}{\gamma }$ being comparable to the vibrational timescale *τ*_*LJ*_ (see “Methods”), which ensures that the dynamics is in the overdamped regime^24^. Equation (1) for the assembly of particles is numerically integrated, using velocity Verlet algorithm, and we monitor the dynamics of density relaxations and time series of energies, stresses etc., by changing *f* at different values of *τ*_*p*_.

We define extreme active matter to be one where (a) the magnitude of the active force is larger than both thermal forces, *fσ*/*k*_*B*_*T* ≫ 1, and the typical force exerted on a particle by the other particles, *fσ*/*ϵ* ≳ 1, and (b) the persistence time *τ*_*p*_ is larger than characteristic relaxation times of the system in the absence of activity. In the athermal limit, which we consider for our study, condition (b) above is replaced by *Pe* ≫ 1, where *Pe* is an active Péclet number defined as *Pe* ≡ *fτ*_*p*_/(*γσ*)^25^.

We now describe the dynamical behaviour observed in the different regions of the phase diagram of Fig. 1.

### Low persistence time—dynamical arrest

**Fig. 2:**
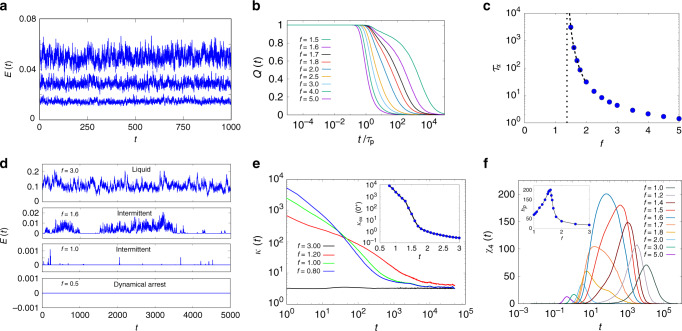
**Dynamical arrest and intermittency at low and intermediate***τ*_*p*_. **a**–**c** Low *τ*_*p*_ = 1: **a** Kinetic-energy time series, *E*(*t*), at different *f* = 2.5, 2.0, and 1.5 (top to bottom), show regular fluctuations; both the mean and the variance reduce with decreasing *f*. **b** Density fluctuations, measured via the self-overlap function *Q*(*t*), relax more slowly as the activity *f* is reduced. **c** The slowness of the relaxation dynamics is measured by the *α*-relaxation time, *τ*_*α*_, extracted from *Q*(*t*), for each *f*. The measured *τ*_*α*_ versus *f* is fitted (dashed line) using a diverging power-law form, which traces out the limit of dynamical arrest *f*_*c*_(*τ*_*p*_) (dotted line) for small *τ*_*p*_ in Fig. 1. **d**–**f** Intermediate *τ*_*p*_ = 10^4^: **d** Kinetic-energy time series as *f* is lowered, shows Gaussian fluctuations at high *f* > *f*^ *^, intermittent bursts and quiescence and finally dynamical arrest when *f* ≤ *f*_*c*_. **e** Intermittency is characterised by the behaviour of the time-dependent kurtosis of the kinetic energy time series, $\kappa (t)=\frac{\langle {(E({t}_{0}\, +\, t)\, -\, E({t}_{0}))}^{4}\rangle }{{\langle {(E({t}_{0}\, +\, t)\, -\, E({t}_{0}))}^{2}\rangle }^{2}}$. We see that in the small *t* end of this log plot, *κ*(*t*) increases linearly as *t* decreases, and should therefore diverge, when extrapolated to *t* → 0. The dynamical order parameter is measured from the value of *κ*(*t*) at the earliest time that we can evaluate, i.e., *t* = 0^+^. (Inset) Variation of the dynamical order parameter, the excess kurtosis, *κ*_*ex*_(0^+^) with *f*. We use the point of inflection of this curve to determine the phase boundary to the intermittent phase. **f** The fluctuation *χ*_4_(*t*) = 〈*Q*^2^(*t*)〉 − 〈*Q*(*t*)〉^2^, shows a peak at a time *t* for different values of *f*. (inset) At a fixed *τ*_*p*_ = 10^4^, the value of the peak height *h*_*p*_ increases sharply as *f* approaches *f*^ *^(*τ*_*p*_) from above, then reduces again. The value of *f* at which *h*_*p*_ has a maximum, for different values of *τ*_*p*_, also marks the boundary between the liquid and the intermittent phase (see Fig. 1).

At high propulsion force, *f*, the material is a fluid with time correlations of density fluctuations measured via the self-overlap function *Q*(*t*) relaxing diffusively (see Fig. 2b; Supplementary Fig. 1). As *f* is reduced, density relaxations slow down, until the onset of glass transition at *f* = *f*_*c*_(*τ*_*p*_), estimated by fitting the variation of the relaxation times *τ*_*α*_ versus *f* (Fig. 2c) with a diverging power law^5^. The glass transition boundary, obtained for a range of *τ*_*p*_ in this low persistence limit, can be fairly accurately described by an active generalisation of the well-known random first-order transition (RFOT) theory^15,17–19^ with an “effective temperature” that goes as *Af*^ 2^*τ*_*p*_/(1 + *Bτ*_*p*_) (see Fig. 1), *A* and *B* being fit parameters^16^. Indeed, recent studies^3–6,10,13^ are consistent with the predictions of this active RFOT theory, in the limit of low *τ*_*p*_. This slowing down of particle motion is also apparent in the time series of the mean kinetic energy *E*(*t*) as one reduces *f*, i.e., the mean and variance of the kinetic energy decrease as *f* → *f*_*c*_(*τ*_*p*_) (Fig. 2a). However, the mean kinetic energy appears to have a nontrivial scaling with *τ*_*p*_ (see Supplementary Fig. 2), over the range of *τ*_*p*_ values investigated. This deviation from equipartition may be due to the fact that the joint distribution of velocities and positions at steady state does not decouple^2,26^.

### Intermediate persistence time—intermittent jamming and plastic yielding

At intermediate persistence times *τ*_*p*_ ≳ 10^3^, the relaxation dynamics is fundamentally different from that at low *τ*_*p*_. At high propulsion force, *f*, the particles move as a fluid as before, with the time series of the mean kinetic energy showing typical Brownian fluctuations (Fig. 2d). As *f* is reduced, the local particle displacements start to show spatial correlations, with a growing correlation length as one approaches *f* = *f*^ *^(*τ*_*p*_) (see Supplementary Fig. 3). At and below the transition *f*^ *^, the average kinetic energy (*E*(*t*)) shows sudden bursts (over a time interval *τ*_1_) with periods of quiescence or jamming (over a time interval *τ*_2_), typical of intermittency^21^, as shown in Fig. 2d, characterised by large fluctuations.

To describe the dynamics of such statistical quantities that alternate between periods of quiescence and large changes over very short times, we monitor their time-dependent fourth moment or kurtosis $\kappa (t)=[\langle {(E({t}_{0}+t)-E({t}_{0}))}^{4}\rangle ]/[\langle (E({t}_{0}+t)\, -E({t}_{0}))^{2}\rangle ^{2}]$^21,27^. At *f* > *f*^ *^(*τ*_*p*_), *κ*(*t*) is nearly flat and close to 3, indicating that the fluctuations are close to Gaussian (Note *κ*(*t*) is calculated from the time series of the mean kinetic energy *E*(*t*), without subtracting out the instantaneous drift of the center-of-mass). For *f* ≤ *f*^ *^(*τ*_*p*_), *κ*(*t*) shows an increase at small *t*, that becomes more pronounced with decreasing propulsion force *f*. We observe that *κ*(*t*) shows a power-law divergence at small *t*, a characteristic signature of intermittency^21,27^.

This allows us to describe the intermittent phase by a dynamical order parameter, the excess kurtosis *κ*_*ex*_(0^+^), of the increment in the kinetic energy over an infinitesimal time interval, which goes from 0 (Gaussian distribution) when *f* > *f*^ *^(*τ*_*p*_) to a finite value (indicative of broad non-Gaussian distributions) across a continuous transition at *f* = *f*^ *^(*τ*_*p*_) (Fig. 2e). The change in this order parameter is sharp for large values of *τ*_*p*_ (inset of Fig. 2e) and becomes more gradual as *τ*_*p*_ is reduced, indicating that at lower *τ*_*p*_, the transition from liquid to intermittent phase is more like a crossover. From the variation of this dynamical order parameter over the {*τ*_*p*_, *f*} plane, we plot the non-equilibrium phase boundary in Fig. 1.

Other quantities begin to show a broad distribution as *f* → *f*^ *^(*τ*_*p*_), such as in the time correlation of density fluctuations, *Q*(*t*). We see this in the fluctuations, *χ*_4_(*t*) = 〈*Q*^2^(*t*)〉 − 〈*Q*(*t*)〉^2^, a measure of the dynamical heterogeneity. At fixed *f*, *χ*_4_(*t*) typically shows a peak at a time *t* (which is less than *τ*_*p*_), as shown in Fig. 2f, and we denote the peak height as *h*_*p*_. The value of *h*_*p*_ increases sharply as *f* approaches *f*^ *^(*τ*_*p*_) from above, then reduces again (see inset of Fig. 2f). The value of *f* at which *h*_*p*_ has a maximum, for different values of *τ*_*p*_ (see Supplementary Fig. 4), provides another marker of the boundary between the liquid and the intermittent phase, indicated by blue circles in Fig. 1.

**Fig. 3:**
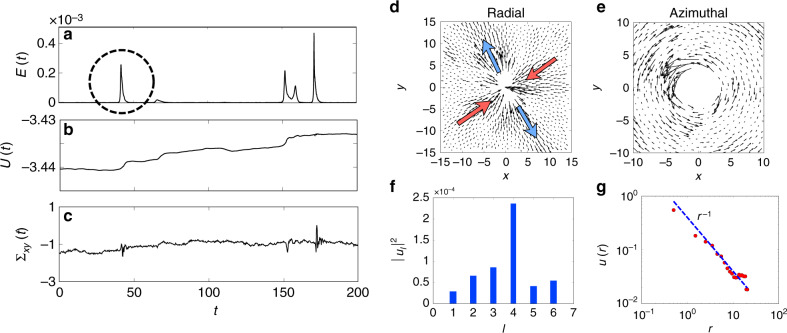
**Intermittent bursts associated with plastic yielding**. *τ*_*p*_ = 10^4^, *f* = 1.0 (marked by green triangle in Fig. 1). We monitor the time series of **a** mean kinetic energy *E*(*t*), **b** potential energy *U*(*t*) and **c** local shear stress *Σ*_*xy*_(*t*), at a value of *f* < *f*^ *^(*τ*_*p*_) where the burst events are isolated and rare. Note that in the kinetic energy time series, we have subtracted out the centre-of-mass contribution. **d**–**g** The displacement field profiles surrounding a single kinetic energy burst event (encircled in **a**): **d** show the radial component of the displacement field with a clear shear axis and quadrupolar symmetry (schematically highlighted using thick arrows), and **e** shows the azimuthal component with signs of vorticity; the arrows in the plots have been scaled up for visibility. **f** The fourfold symmetry shows up as a dominant *l* = 4 mode in the power spectrum of the radial component of the displacement field $u(r,\theta )={\sum }_{l}{u}_{l}(r)\exp (-il\theta )$, where we average over *r* for better signal to noise. **g** Spatial profile of the radial component of the displacement shows a 1/*r* fall from the event. This implies that the deformation associated with a single, isolated yield event is an Eshelby deformation.

Within the intermittent phase, we notice that the sudden increase in kinetic energy during a burst is instantaneously accompanied by a non-reversible release in the potential energy (Fig. 3b), as well as visible spikes in the local shear stress, Fig. 3c (see “Methods” for definition). Thus, driven by persistent active stresses, configurations of particles in the intermittent phase experience a buildup of the elastic stress, a transient jamming (*E* = 0), followed by sudden yielding, seen as a burst of kinetic energy (Supplementary Movies).

The bursts in kinetic energy are accompanied by local structural reorganisations associated with sudden collective non-affine displacements of a finite fraction of particles (see Supplementary Movie 1). These bursty features are apparent in the thresholded displacements over a time *τ* (Supplementary Fig. 5), and more directly in the spatial maps of the particle displacements. This implies that the intermittent steady state exhibits a continual yielding and jamming of macroscopically large structures.

Qualitatively similar intermittent behaviour has been observed in the dynamics of a particle driven through a bath of active discs^28^, in a system of active discs moving in a disordered array of fixed obstacles^29^, and in dense granular flow composed of self-propelled frictional hard particles^30^.

### Plastic yielding—Eshelby deformations

Deep in the intermittent phase, these bursts in kinetic energy and associated plastic yielding, are rare and isolated (Fig. 3a), allowing us to analyse the deformation field around a single burst event.

The radial and azimuthal components of the displacement field **u** (*r*, *θ*) surrounding the single yielding event, show a quadrupolar symmetry (Fig. 3d–f) and a long-range decay with radial distance that goes as 1/*r* (Fig. 3g). A similar feature is shown by the local elastic shear stress propagated as a consequence of a single yielding event. This is the well-studied Eshelby deformation profile^20^, which describes elementary local deformations in an amorphous solid under external uniform shear^31,32^. The unexpected appearance here of the Eshelby stress is a result of local shear arising from internal stirring at the scale of the active particle.

**Fig. 4:**
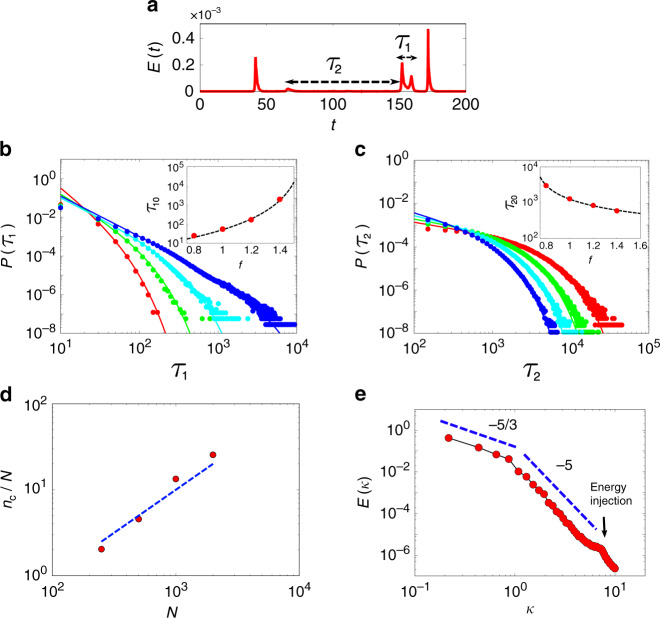
**Statistics of yielding, emergence of viscoelasticity and plastic turbulence**. **a** Within the intermittent phase, bursts of activity having duration *τ*_1_ occur amidst quiescent states having duration *τ*_2_, for *f* = 1.0. **b**, **c** The distributions of the periods of intermittent bursts [shown in **b**] and quiescence [shown in **c**] can be fit to a power law with an exponential cut-off, $P({\tau }_{1}) \sim {\tau }_{1}^{-\alpha }\exp (-{\tau }_{1}/{\tau }_{10})$ and $P({\tau }_{2}) \sim {\tau }_{2}^{-\beta }\exp (-{\tau }_{2}/{\tau }_{20})$, with the corresponding fit functions shown as lines. Data shown for *f* = 0.8 (red), 1.0 (green), 1.2 (cyan) and 1.4 (blue); the exponents vary with *f* and are measured to be *α* = 2.97, 2.30, 2.04 and 2.07 in **b**, *β* = 0.57, 0.57, 0.72 and 0.94 in (**c**). The inset shows the variation of the cut-offs *τ*_10_ and *τ*_20_ with *f*. We note that the cut-off for *τ*_1_, moves to larger times as *f* → *f*^ *^(*τ*_*p*_) from below, whereas *τ*_20_ increases as *f* → *f*_*c*_(*τ*_*p*_) from above, giving rise to a power-law behaviour at the two limits. **d** The intermittent yielding events involve the non-affine displacement of a finite fraction of particles, as seen in this plot of *n*_*c*_, the number of particles that show a non-affine displacement within a time window Δ*t* = 10^4^, versus total particle number *N*, where the dashed line has slope 1. **e** The scale-free intermittency close to the phase boundary *f*^ *^(*τ*_*p*_), is associated with plastic turbulence as seen in the spectrum of the energy density *E*(*k*) that shows an inverse cascade from an injection scale, shown by the arrow. Data shown for *f* = 1.6, *τ*_*p*_ = 10^4^. The crossover from a steep spectrum *k*^−5^ to the Kolmogorov spectrum *k*^−5/3^ at lower *k*, is set by the scale of the vorticity.

As one moves towards the intermittent phase–liquid boundary from below, the bursts get more frequent and are bunched up. The distributions of the periods of intermittent bursts (*τ*_1_) and quiescence (*τ*_2_) (see Fig. 4a for definition), are power laws with exponential cut-offs that depend on *f*: $P({\tau }_{1}) \sim {\tau }_{1}^{-\alpha }\exp (-{\tau }_{1}/{\tau }_{10})$ and $P({\tau }_{2}) \sim {\tau }_{2}^{-\beta }\exp (-{\tau }_{2}/{\tau }_{20})$; see Fig. 4b, c. The cut-off *τ*_10_ increases as *f* approaches *f*^ *^(*τ*_*p*_) from below; in the vicinity of *f*^ *^(*τ*_*p*_), *P*(*τ*_1_) appears scale-invariant, going as $\approx {\!}{\tau }_{1}^{-2.07}$. On the other hand, the cut-off *τ*_20_ increases as *f* approaches *f*_*c*_(*τ*_*p*_) from above.

Each of these plastic events gives rise to stresses that propagate through the material (see Supplementary Movies 2 and 3). Outside the plastic zones, the rest of the material should respond elastically, with an anisotropic elastic kernel. However, the occurrence of multiple yielding events will result in strong correlations between events, one triggering another, that will make the kernel isotropic, since the directions of local shear due to active forcing would be randomly oriented.

### Accumulated yielding and turbulence

Since, for a small enough *f*, the plastic bursts are bunched-up discrete events, the number of particles *n*_*c*_(Δ*t*) that undergo irreversible displacement within a time window Δ*t* is the number of displaced particles per event times the number of events within the window Δ*t*. We find that *n*_*c*_ ~ *N*^2^ (Fig. 4d), suggesting that each intermittent yielding event involves the collective displacement of a finite fraction of particles^33^. This implies that the occurrence of more and more of such events will cause the material to flow at long timescales, with a time-dependent viscosity *η*(*t*), determined by the accumulated yielding upto time *t*, eventually reaching a constant steady-state value for *t* ≫ *τ*_*p*_. This shows up, at the level of single tagged-particle dynamics, as eventual diffusive motion when *t* ≫ *τ*_*p*_ (see Supplementary Figs. 6 and 8), and coincides with the relaxation of the self-overlap function *Q*(*t*).

As we reduce the active force *f*, the tagged-particle diffusion coefficient decreases (Supplementary Fig. 8), and eventually vanishes as one approaches dynamical arrest. As before, we obtain the dynamical arrest boundary *f* = *f*_*c*_(*τ*_*p*_) (see Fig. 1), by fitting the data for the *α* − relaxation time *τ*_*α*_ (Supplementary Fig. 7) measured from *Q*(*t*), to a power-law divergence. As shown in Supplementary Fig. 9, the value of *f*_c_(*τ*_*p*_) remains finite in the thermodynamics limit.

On the other hand, as we increase the forcing *f* towards the phase boundary *f*^ *^(*τ*_*p*_), we observe that the scale-free intermittency displays a kind of plastic turbulence^34,35^ in an actively stirred dense material. This is seen in the spatiotemporal dynamics of the displacement fields that show large swirls, see Supplementary Movie 4, and in the mean kinetic-energy density or the power spectrum of the velocity fluctuations. The intermittent jamming–yielding due to local active stirring transfers energy from small scales and dissipates it over larger scales, leading to an inverse cascade, where the energy spectrum crosses over from *E*(*k*) ~ *k*^−5^ to *E*(*k*) ~ *k*^−5/3^ at lower *k* (Fig. 4e). The crossover to the Kolmogorov spectrum happens at a scale corresponding to the scale of vorticity^36^. This stress production and dissipation gives rise to a non-equilibrium steady state with a finite energy–current.

### Infinite persistence—jamming/unjamming

**Fig. 5:**
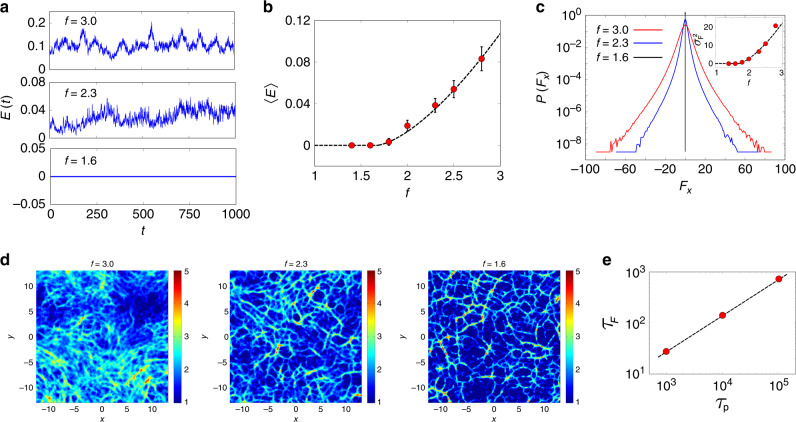
**Jamming at infinite***τ*_*p*_**and force chains**. **a** Kinetic-energy time series as *f* is lowered, shows complete jamming at *f* ≈ 1.6. **b** The variation of the mean kinetic energy with *f*, shows a continuous transition at *f* = *f*^ *^(*∞*) ≈ 1.6, that goes as 〈*E*〉 ~ ∣*f* − *f*^ *^(*∞*)∣^3/2^, shown with dashed line. Error bars denote a standard deviation over 32 independent initial conditions. **c** The probability distribution of the *x* component of the total force acting on a particle (passive LJ + active forces), *P*(*F*_*x*_) at different values of *f*, is broad with exponential tails, with a width that decreases continuously with *f* (inset). From each particle, we have subtracted the centre-of- mass force. At the jamming transition, *f* = *f*_*c*_, the distribution becomes a delta function at *F*_*x*_ = 0, the force-balanced state. **d** At the jamming critical point, the forces on the particles are distributed along force chains, as highlighted in the colour map of thresholded forces. The forces are evaluated using a local coarse graining of inter-particle forces acting along each bond between local neighbours, time averaged over *δt* = 5. The colour represents the strength or magnitude of the forces (shown in the colour bar). Away from the jamming critical point, either by increasing *f* keeping *τ*_*p*_ fixed, or decreasing *τ*_*p*_ keeping *f* fixed, the force chains dynamically remodel whilst still being embedded in a static contact network. This is seen as a blurring of the colour map of thresholded forces (at *f* = 3.0 and 2.3) away from the sharp force chains at *f* = 1.6. **e** The dynamics of the force chains show a distribution of lifetimes in the force-balanced configuration. The mean lifetime of the force-balanced configurations, computed for *f* = 1.6 at varying *τ*_*p*_, diverges as one moves towards the jamming critical point as a power law, ${\tau }_{F} \sim {\tau }_{p}^{z}$, with *z*  = 0.71 (shown with dashed line).

Analysis of the *τ*_*p*_ = *∞* limit, brings in a new facet of extreme active matter. This limit corresponds to a situation where the initial directions of particle self-propulsion are quenched in random directions. From being a fluid with mobile particles at large *f*, the assembly jams at *f*^ *^(*∞*) ≃ 1.67, where the kinetic energy goes to zero as  ~ ∣*f* − *f*^ *^(*∞*)∣^3/2^ (Fig. 5a, b). The distribution of the total force (LJ + active forces) *P* (**F**) changes from a broad distribution with exponential tails to a delta function at **F** = 0 at *f*^ *^(*∞*); the jamming transition is associated with a force-balanced configuration of the soft particles (Fig. 5c). As shown in the inset of Fig. 5c, the width of *P*(**F**) and the mean kinetic energy go continuously to zero as *f* approaches *f*^ *^(*∞*) (on the other hand, the broad tails are highlighted by plotting the distribution of the force scaled by its root-mean-square value, see Supplementary Fig. 10). This allows us to identify *f*^ *^(*∞*) as a jamming/unjamming force threshold for active yielding. As discussed in a recent work^37^, the density-dependent *f*^ *^(*ρ*, *∞*) will trace out a yielding line in the jamming-phase diagram^38^ of dense amorphous materials, with active forcing being the control variable. Thus, we expect to find critical behaviour in the proximity of *f*^ *^(*∞*).

In the vicinity of *f*^ *^(*∞*), we map the contact network of particles, and evaluate the net interaction force between pairs of soft particles in contact; we find that these forces are distributed along force chains (see plot for *f* = 1.6 in Fig. 5). With increasing *f*, the force chains dynamically remodel, as the structures relax; see plots for *f* = 2.3, 3.0 in Fig. 5d. Likewise, dynamical reorganisation of the force network also occurs when we move slightly away from the jammed regime, by decreasing *τ*_*p*_, keeping *f* fixed. Under these conditions, the dynamics of force chains typically show periods of jamming in a force-balanced configuration, interspersed with bursts of remodelling (Supplementary Movie 5). As one approaches the jamming threshold at *τ*_*p*_ = *∞*, the mean lifetime of the force-balanced configurations diverges as ${\tau }_{F} \sim {\tau }_{p}^{z}$, with a new dynamical critical exponent *z* ≈ 0.71 (Fig. 5e).

We point to an interesting non-monotonic feature in Fig. 1—for a range of values of *f* near *f* = 1, the system goes from a dynamically arrested state to a liquid and then back to a jammed state, as *τ*_*p*_ changes from 0 to *∞*.

## Discussion

Extreme active matter at high densities brings together the physics of glass, jamming, plasticity and turbulence, in a new state of driven classical matter. As we have seen, tuning the persistence time *τ*_*p*_ enables us to explore the crossover between glass physics, where the dynamics proceeds by density relaxation, and jamming-yielding physics, where the dynamics is controlled by stress buildup and release via macroscopic flows. We emphasise that the intermittent plastic deformation and turbulent flows are constitutive and do not respond to an externally imposed stress.

While our present study was done at *T* = 0, we have checked that including a small temperature via a thermal noise *ϑ*_*i*_ gives similar results, as long as the active Péclet number is high; the crossover behaviour from these different regimes is likely to be quite subtle.

To show that the dynamical regimes discussed here do not crucially depend on inertia present in the translational dynamics in Eq. (1), we have also studied the dynamics of an equivalent active glass model (binary WCA mixture^39^) in the fully overdamped limit. We find that our observations remain unchanged, and all the dynamical regimes reported in Fig. 2 are found to be present (Supplementary Fig. 11).

We have fixed the overall density of particles in our study; however, Liao and Xu^37^ show that the athermal jamming transition at *τ*_*p*_ = *∞* occurs over a range of densities, making this active jamming critical point density dependent. If however we make the density very low, while still keeping *τ*_*p*_ = *∞*, we would arrive at a jammed gas phase, with isolated islands of jammed material^25^ (Chakrabarty B., private communication).

Are there natural or synthetic realisations of extreme active matter? Herds of animals, such as penguins or bulls, dense collection of vehicles, ants or microbots and even trite examples such as a scrum of rugby players, could be possible realisations. Promising candidates for extreme active matter are monolayers of persistently motile cells; indeed, Garcia et al.^40^ observe jamming-yielding behaviour in such monolayers of epithelial cells. A model similar to the one considered here has been used in a recent study^26^ of motion patterns in confluent cell monolayers. Recent experiments^41,42^ on dense systems of Janus colloids have provided a physical realisation of dense active matter near the glass transition. It would be challenging to construct synthetic realisations of extreme active matter, and we eagerly look forward to controlled experimental studies on these.

## Methods

### Models

We discuss the details of the model system, and the implementation of the numerical integration scheme for Eq. (1). Our model is the well-studied two-dimensional Kob–Anderson binary mixture^22^, comprising particles A, B in the ratio 65:35, interacting via a Lennard–Jones (LJ) potential

2 $${V}_{ij}(r)=4{\epsilon }_{\alpha \beta }\left[{\left(\frac{{\sigma }_{\alpha \beta }}{r}\right)}^{12}-{\left(\frac{{\sigma }_{\alpha \beta }}{r}\right)}^{6}\right]$$

where *α*, *β* ∈ {*A*, *B*}, *r* is the distance between the *i* and *j* particles *r* = ∣**r**_*i*_ − **r**_*j*_∣. The strength and range of the interaction are set by *ϵ*_*αβ*_ and *σ*_*αβ*_, respectively, where we fix *σ*_*AB*_ = 0.8, *σ*_*BB*_ = 0.88, *ϵ*_*AB*_ = 1.5, and *ϵ*_*BB*_ = 0.5, in units where *σ*_*AA*_ = *ϵ*_*AA*_ = 1. The composition of the *A*:*B* mixture helps to avoid crystallisation in the absence of activity. Throughout our study, we fix the overall number density to be *ρ* = 1.2, which is in the regime where the passive model shows dynamic arrest at low temperatures. All particles have the same mass (*m* = 1) and the LJ time unit is ${\tau }_{LJ}\equiv \sqrt{m{\sigma }_{AA}^{2}/{\epsilon }_{AA}}=1$.

### Simulation details

The number of particles used in the simulation varies between *N* = 1000–10,000. All data presented here have also been averaged over 32–96 independent realisations, unless mentioned otherwise. We note here that statistically the time-averaged net force 〈∑_*i*_ *f***n**_*i*_〉 is zero. However, the instantaneous net force can be non-zero, and thus for computing dynamical quantities of interest, this instantaneous drift of the system’s centre of mass is subtracted out (unless stated otherwise).

### Dynamical quantities

To characterise the dynamics of the dense liquid under steady state or transient conditions, as we vary the active forcing (*f*) for various choices of *τ*_*p*_, we measure the mean-squared displacement (MSD) Δ^2^(*t*) and self-part of the two-point overlap correlation function *Q*(*t*), defined as

3 $${\Delta }^{2}(t)=\left\langle \frac{1}{N}\sum _{i}| {{\bf{r}}}_{i}({t}_{0})-{{\bf{r}}}_{i}(t+{t}_{0}){| }^{2}\right\rangle$$

4 $$Q(t)=\left\langle \frac{1}{N}\sum _{i}q(| {{\bf{r}}}_{i}({t}_{0})-{{\bf{r}}}_{i}(t+{t}_{0})| )\right\rangle$$

where

5 $$q(r)=\left\{\begin{array}{ll}1&\,\hskip -10pt \text{if}\,r\, \le \, b\\ 0&\,\text{otherwise}\,\end{array}\right.$$

〈 ⋯ 〉 represents an average over the time origin *t*_0_, *N* is the number of particles in the system and the parameter *b* is associated with the typical vibrational amplitude of the caged particles. Throughout our analysis, we have used *b* = 0.3, and we have verified that our results are insensitive to moderate changes in *b*.

### Calculation of stress

Since we work in the athermal limit, we calculate the instantaneous volume-averaged virial stress tensor Σ_*αβ*_

6 $${\Sigma }_{\alpha \beta }=-\frac{1}{2\Omega }\sum _{i,j}({r}_{i}^{\alpha }-{r}_{j}^{\alpha })\ {f}_{ij}^{\beta }$$

where ${r}_{i}^{\alpha }$ is the *α*-component of the position of particle *i* and ${f}_{ij}^{\beta }$ is the *β*-component of the interaction force on *i* due to particle *j*. The sum is over all particles *i* and *j* that interact with each other within an interaction volume Ω. This can be defined locally, and the time series of the local Σ_*xy*_(*t*) is shown in Fig. 3c.

The spatially coarse-grained stress field Σ_*αβ*_(**r**, *t*) is obtained as follows: divide the simulation area into a 50- × 50-square grid, then compute the mean stress tensor located at the centroid of each square, by applying an exponential interpolation function on the local stress Σ_*αβ*_ evaluated at all points that lie within the square. The spatial dynamics of the coarse-grained Σ_*xy*_(**r**, *t*) is displayed in Supplementary Movies 2 and 3.

## Supplementary information

**Supplementary Information**

**Description of Additional Supplementary Files**

**Supplementary Movie 1**

**Supplementary Movie 2**

**Supplementary Movie 3**

**Supplementary Movie 4**

**Supplementary Movie 5**

## Data Availability

Data that support the findings are available from the corresponding author upon request.
