# Supplementary material for: Extreme active matter at high densities
Source: Nat Commun. 2020 May 22;11:2581. doi: 10.1038/s41467-020-16130-x (PMC7244575; doi:10.1038/s41467-020-16130-x)
Supplement: Supplementary file 1 — Supplementary Information [file 41467_2020_16130_MOESM1_ESM.pdf]

Supplementary Information:
Extreme active matter at high densities

Rituparno Mandal,[1,*] Pranab Jyoti Bhuyan,[2,†] Pinaki Chaudhuri,[3,‡] Chandan Dasgupta,[2,4,§] and Madan Rao[1,¶]

[1] *Simons Centre for the Study of Living Machines, National Centre for Biological Sciences (TIFR), Bangalore 560065, India*
[2] *Centre for Condensed Matter Theory, Department of Physics, Indian Institute of Science, Bangalore 560012, India*
[3] *The Institute of Mathematical Sciences, Chennai 600113, India*
[4] *International Centre for Theoretical Sciences (TIFR), Bangalore 560089, India*

I. SUPPLEMENTARY FIGURES

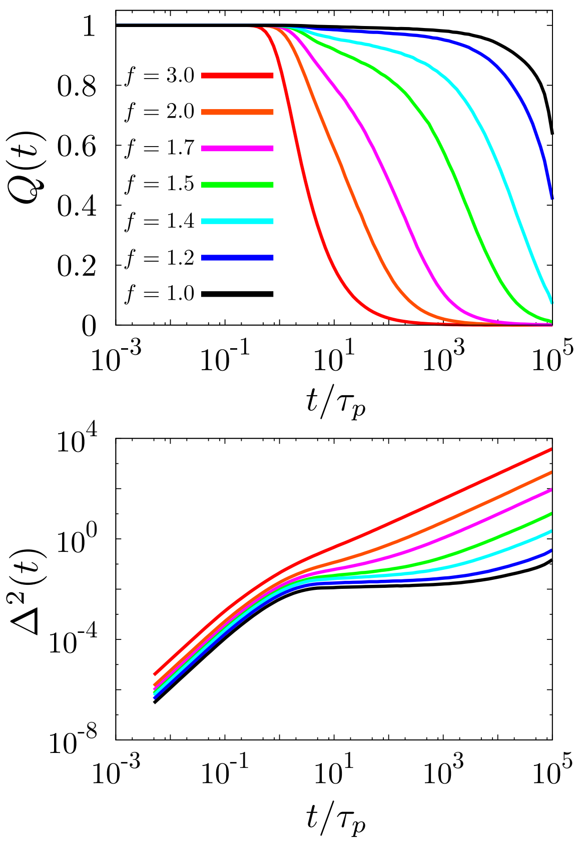

Supplementary Figure 1. $\tau_p = 1$. (Top) Self-overlap function, $Q(t)$, for different values of active forcing f, as indicated. (Bottom) Corresponding mean squared displacement, $\Delta^2(t)$. Both quantities show that relaxation timescales increase with decreasing f.

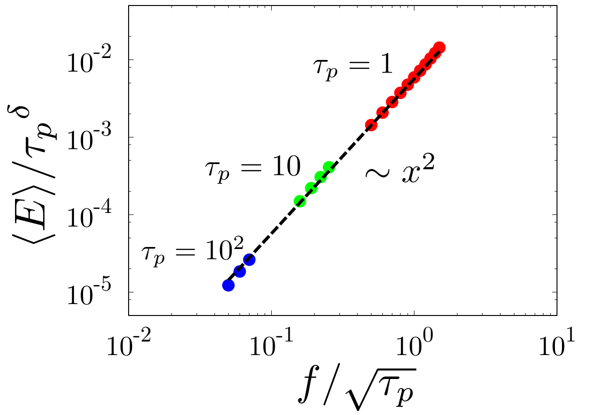

Supplementary Figure 2. Dependence of mean kinetic energy on f and τ_p, shown here as a scaling plot, $\langle E \rangle \propto \tau_p^{\delta} G(f \tau_p^{-\alpha})$, where $\delta = 0.11$, $\alpha = 0.5$, for values of $1 \leq \tau_p \leq 100$. For smaller values of τ_p, one might expect a crossover.

* Email: rituparno@ncbs.res.in
† Email: pranab@physics.iisc.ac.in
‡ Email: pinakic@imsc.res.in
§ Email: cdgupta@iisc.ac.in
¶ Email: madan@ncbs.res.in

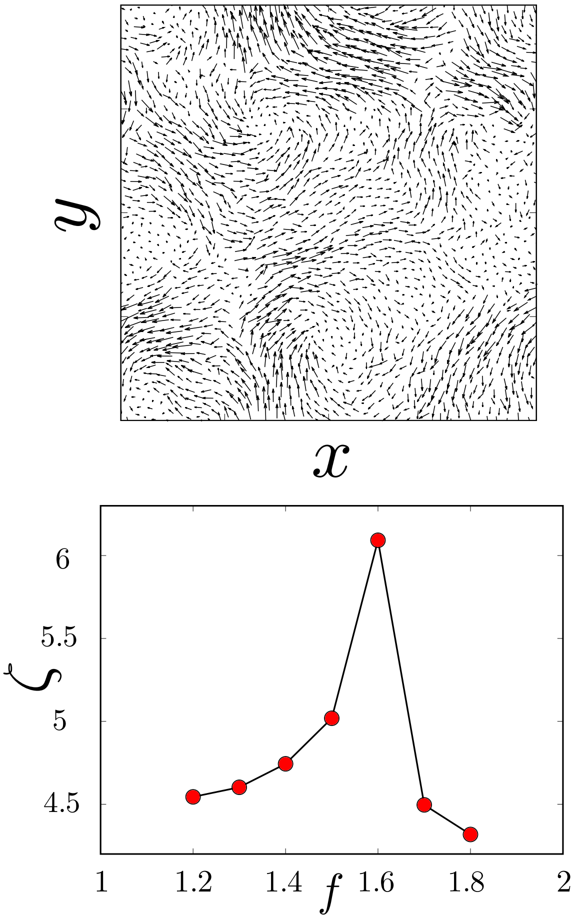

Supplementary Figure 3. $\tau_p = 10^4$. (Top) A typical displacement field map (calculated over the density relaxation timescale τ_α) at $f = 1.4$, showing strong spatial correlations, and the emergence of swirl-like collective motion during $\alpha-$relaxation time scale. (Bottom) With changing f, variation of dynamical length scale, ζ, calculated from spatial correlation function $C(r) = \langle \vec{x}_{\tau_\alpha}(\vec{r}) \cdot \vec{x}_{\tau_\alpha}(\vec{0}) \rangle$ where $\vec{x}_{\tau_\alpha}(\vec{r})$ is the displacement of the particles at position $\vec{r}$ over the timescale τ_α. To extract the length scale (ζ) associated with the size of this cooperatively rearranging regions we use the relation $C(\zeta)/C(0) = 1/e$. ζ peaks at the transition region ($f \sim 1.6$) between the transition from the intermittent regime to the liquid-like regime.

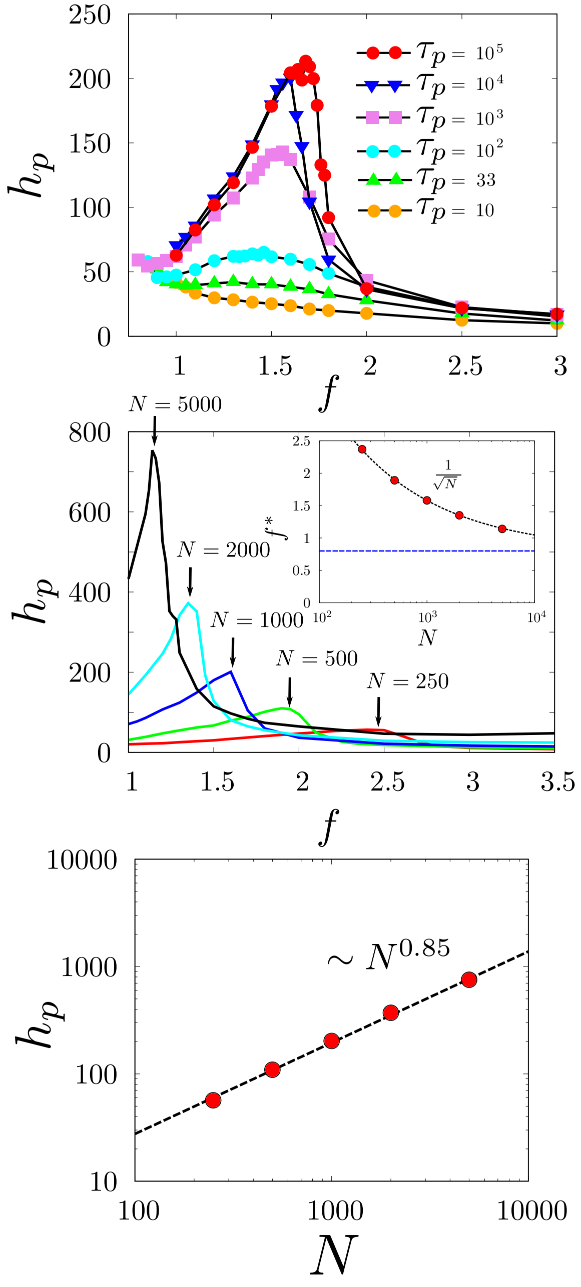

Supplementary Figure 4. (Top) Variation of peak height (h_p) of $\chi_4(t)$, the fluctuation of the overlap function $Q(t)$, with active forcing f, for various values of τ_p, shows non-monotonic behaviour in the range $\tau_p > 10$. The locus of f at which h_p has a maximum, for each τ_p, defines the boundary between intermittent and fluid regimes. (Middle) The variation of h_p with f, for various system sizes, shows that the fluctuations increase with increasing system size N, indicating an underlying dynamical transition. The inset shows that f^*, the force at which the peak occurs, remains finite in the thermodynamic limit and the horizontal dashed line shows the value of f^* extrapolated to infinite N. (Bottom) The dependence of h_p, the peak height of the four-point susceptibility χ_4, on the system size N for $\tau_p = 10^4$. The black dashed line is the best fit to a power law.

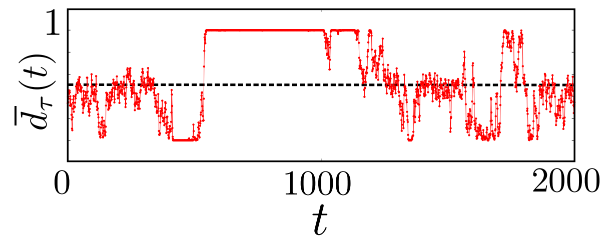

Supplementary Figure 5. $\tau_p = 10^4$. In the intermittent phase (and in the vicinity of the liquid-intermittent boundary), the system switches between a jammed and flowing region, as captured by the displacement overlap function $\bar{d}_\tau(t)$, averaged over all particles, and defined as $d_\tau(t) = 1$ if the displacement between time $t - \tau$ and t is more than a and $d_\tau(t) = 0$ if it is smaller than a, where we chose $a = 0.1$ and $\tau = 1.0$. Here, the data is shown for propulsion force $f \sim 1.6$. The dashed line corresponds to 0.5.

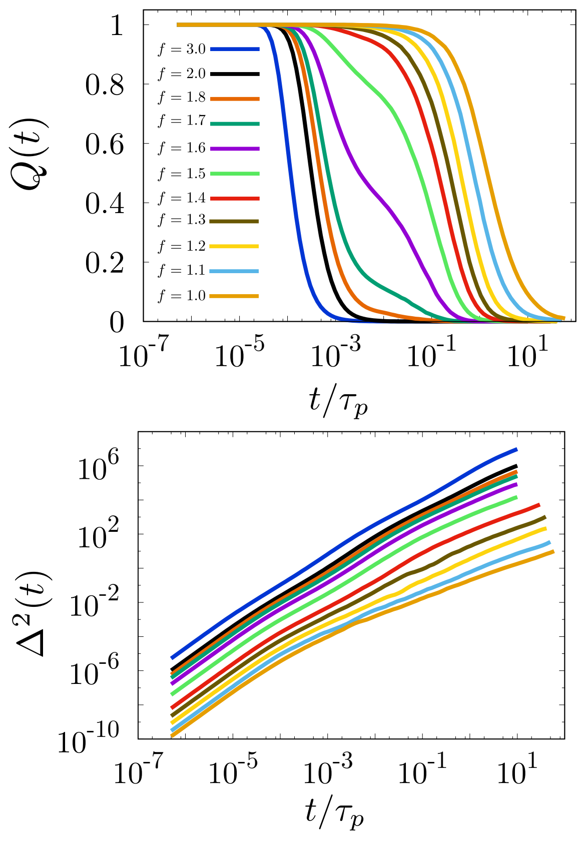

Supplementary Figure 6. $\tau_p = 10^4$. (Top) Self-overlap function, $Q(t)$, for different values of active forcing f, as indicated. (Bottom) Corresponding mean squared displacement, $\Delta^2(t)$. The relaxation functions show a change in behaviour around $f = 1.6$, which is where the relaxation timescale τ_α shows a jump (see Supplementary Figure 7) and the peak value of $\chi_4(t)$ shows a maximum, with changing f, as shown in Supplementary Figure 4.

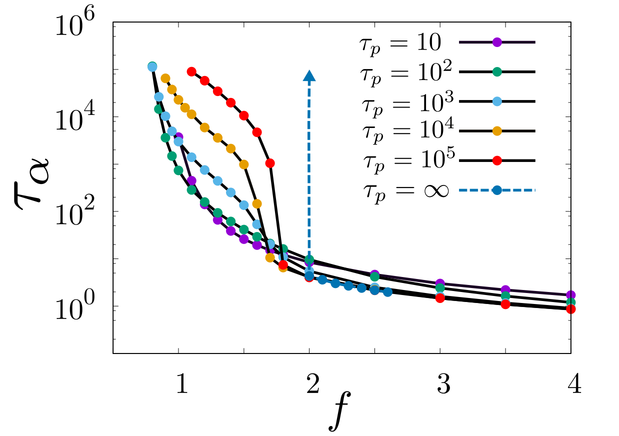

Supplementary Figure 7. Relaxation timescale τ_α extracted from $Q(\tau_\alpha) = 1/e$, as a function of f for a range τ_p (see labels). For $\tau_p > 10$, τ_α vs f has a jump, with the location corresponding to where there is a peak in h_{χ_4}, as shown in Supplementary Figure 4.

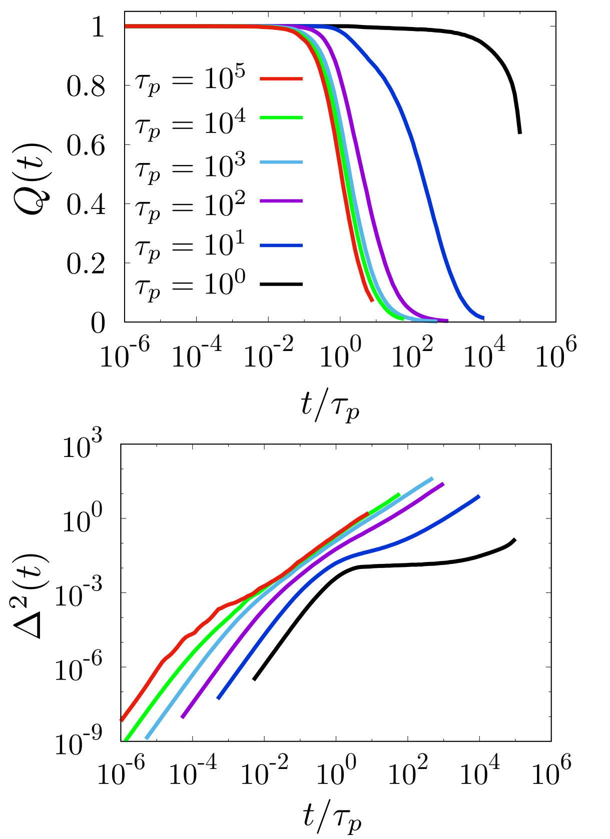

Supplementary Figure 8. $f = 1$. (Top) Self-overlap function, $Q(t)$, for different values of persistence time τ_p of self-propulsion, as indicated. (Bottom) Corresponding mean squared displacement, $\Delta^2(t)$. For $\tau_p \geq 10^3$, the characteristic relaxation timescale is $t/\tau_p \approx 1$, and diffusive motion is also seen to set in, beyond this timescale.

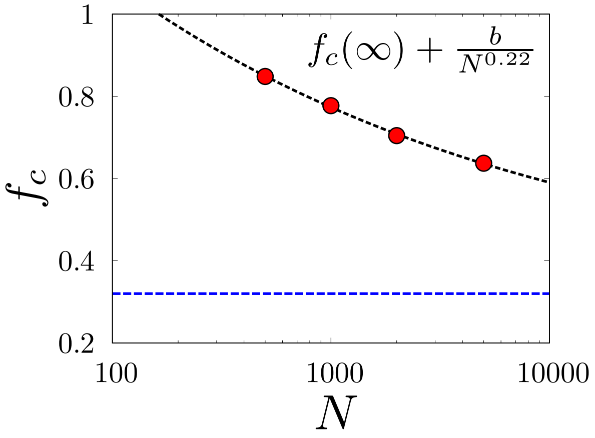

Supplementary Figure 9. The dependence of f_c, the value of f corresponding to the transition from the intermittent liquid to the dynamically arrested phase, on the system size N for $\tau_p = 10^4$. The black dashed line is a fit to the form $f_c(N) = f_c(\infty) + b/N^{0.22}$ and the blue dashed line shows the value of $f_c(\infty)$.

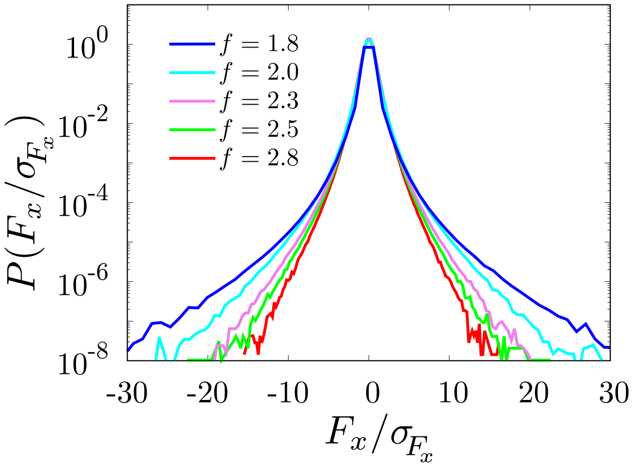

Supplementary Figure 10. The probability distribution of F_x, the x-component of the total force acting on a particle, scaled by its root-mean-square value σ_{F_x}, for different values of f in the $\tau_p \to \infty$ limit. This plot highlights the broad tails present in the force distribution.

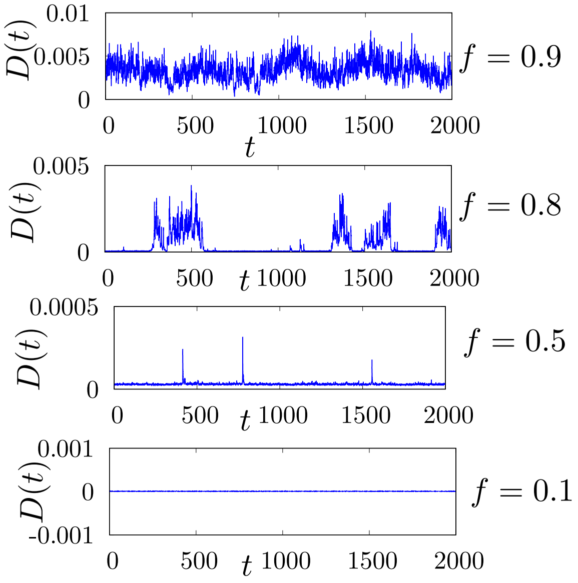

Supplementary Figure 11. Fully overdamped dynamics of an equivalent active glass model (binary WCA mixture[a]), where only the repulsive part of the Lennard-Jones interaction is present. The mobility $D(t)$, is defined as the root mean square of the displacement during times t and $t+\Delta t$, averaged over all particles ($\Delta t = 1$). The parameter values are, $\tau_p = 10^4$, $N = 1000$, $\rho = 1.2$, $T = 0$. Results for four different values of f between 0.1 and 0.9 are shown. We find that all the dynamical regimes reported in Fig. 2 of the main text, are observed here too.

[a] L. Berthier and G. Tarjus, The role of attractive forces in viscous liquids, *J. Chem. Phys.* **134**, 214503-214512 (2011)
