# Supplementary material for: Extreme active matter at high densities
Source: Nat Commun. 2020 May 22;11:2581. doi: 10.1038/s41467-020-16130-x (PMC7244575; doi:10.1038/s41467-020-16130-x)
Supplement: Supplementary file 2 — Description of Additional Supplementary Files [file 41467_2020_16130_MOESM2_ESM.pdf]

## Description of Additional Supplementary Files

1. Supplementary Movie 1 (*1intermittency.avi*): The intermittent bursts in the time series of the mean kinetic energy (below), and corresponding thresholded displacement field showing localised events. Here, the self-propulsion force $f = 1$, and the persistence time $\tau_p = 10^4$.

2. Supplementary Movie 2 (*2compress.avi*): For $\tau_p = 10^4$ and $f = 1$, we observe propagation of compressive stresses, following a structural rearrangement event, marked by a spike in the kinetic energy.

3. Supplementary Movie 3 (*3shear.avi*): For $\tau_p = 10^4$ and $f = 1$, we observe propagation of shear stresses, following a structural rearrangement event, marked by a spike in the kinetic energy.

4. Supplementary Movie 4 (*4swirl.avi*): For $\tau_p = 10^4$, $f = 1.4$, we observe swirling patterns in the displacement field, interspersed with periods of quiescence. The corresponding time series of kinetic energy (shown below) shows bursts of activity corresponding to the periods of swirl motion.

5. Supplementary Movie 5 (*5forcechain.avi*): For $\tau_p = 10^4$, $f = 1.6$, (top-left) shows the dynamic remodelling of force chains, corresponding to (top-right) the evolution of the particle positions. (Below left) shows the time series of mean potential $U(t)$ and kinetic $E(t)$ energies. (Below right) shows the time evolution of $P(F_x)$, the distribution of the x-component of the force acting on each particle, which shows periods of jamming and force-balance with $P(F_x) \approx \delta(F_x)$, followed by bursts of activity corresponding to a continuously remodelling force network with $P(F_x)$ showing broad tails.
